# The effect of growth on the correlation between the spinal and rib cage deformity: implications on idiopathic scoliosis pathogenesis

**DOI:** 10.1186/1748-7161-2-11

**Published:** 2007-09-14

**Authors:** Theodoros B Grivas, Elias S Vasiliadis, Constantinos Mihas, Olga Savvidou

**Affiliations:** 1 Orthopaedic Department, "Thriasio" General Hospital, G. Gennimata Av. 19600, Magoula, Attica, Greece

## Abstract

**Background:**

Numerous studies have attempted to quantify the correlation between the surface deformity and the Cobb angle without considering growth as an important factor that may influence this correlation. In our series, we noticed that in some younger referred children from the school-screening program there is a discrepancy between the thoracic scoliometer readings and the morphology of their spine. Namely there is a rib hump but no spinal curve and consequently no Cobb angle reading in radiographs, discrepancy which fades away in older children. Based on this observation, we hypothesized that in scoliotics the correlation between the rib cage deformity and this of the spine is weak in younger children and vice versa.

**Methods:**

Eighty three girls referred on the basis of their hump reading on the scoliometer, with a mean age of 13.4 years old (range 7–18), were included in the study. The spinal deformity was assessed by measuring the thoracic Cobb angle from the postero-anterior spinal radiographs. The rib cage deformity was quantified by measuring the rib-index at the apex of the thoracic curve from the lateral spinal radiographs. The rib-index is defined as the ratio between the distance of the posterior margin of the vertebral body and the most extended point of the most projecting rib contour, divided by the distance between the posterior margin of the same vertebral body and the most protruding point of the least projecting rib contour. Statistical analysis included linear regression models with and without the effect of the variable age. We divided our sample in two subgroups, namely the younger (7–13 years old) and the older (14–18 years old) than the mean age participants. A univariate linear regression analysis was performed for each age group in order to assess the effect of age on Cobb angle and rib index correlation.

**Results:**

Twenty five per cent of patients with an ATI more than or equal 7 degrees had a spinal curve under 10 degrees or had a straight spine. Linear regressions between the dependent variable "Thoracic Cobb angle" with the independent variable "rib-index" without the effect of the variable "age" is not statistical significant. After sample split, the linear relationship is statistically significant in the age group 14–18 years old (p < 0.03).

**Conclusion:**

Growth has a significant effect in the correlation between the thoracic and the spinal deformity in girls with idiopathic scoliosis. Therefore it should be taken into consideration when trying to assess the spinal deformity from surface measurements. The findings of the present study implicate the role of the thorax, as it shows that the rib cage deformity precedes the spinal deformity in the pathogenesis of idiopathic scoliosis.

## Background

Numerous studies have attempted to quantify the correlation between the surface deformity and the Cobb angle in patients with idiopathic scoliosis (IS), [1-10].

Several non-invasive methods have been introduced and mathematical models have been developed, all attempting to predict the Cobb angle from the surface deformity. The use of scoliometer, the Moire topography, the Integrated Shape-Imaging System (ISIS) [8], the more advanced 360° torso scanners, [4,11] and the artificial neural networks (ANNs) [12] are examples of such attempts. All have been developed because repeated radiographs which are currently used for scoliotic patients' follow up can increase the risk of cancer as a consequence of increased ionizing radiation [13].

In a recent report Bunnel states that "It has become apparent from many reports that although there is a significant correlation between clinical deformity and radiographic measurement, the standard deviation is so high that it is not possible to reliably predict the degree of curvature from surface topography in any given patient by any technique" [14,5-18,9]. Bunnel also states that, in general, clinical deformity is disproportionately greater than expected for the degree of Cobb angle in the early stages of the development of scoliosis [14].

Although IS is considered a lateral curvature of the spine with concordant vertebral rotation [19], asymmetry involves some other structures like the rib cage, the muscles, the viscera, the fat and the skin in a manner that is unique to each patient and changes over time as the deformity progresses [12]. It is interesting that most of the studies that correlate the surface deformity with the Cobb angle are quantifying this correlation without looking into other elements of the torso asymmetry and their possible aetiologic implications.

While the surface deformity appears to correlate with spinal deformity in most of the studies, we monitored that in some younger referred children from school screening there is a discrepancy between the thoracic scoliometer readings and the Cobb angle [20]. Although these children had a notable thoracic asymmetry, they were found to have straight spines in their standing posteroanterior spinal radiographs. Based on the above observation, we hypothesized that in scoliotics the correlation between the rib cage deformity and this of the spine is weak in younger children and vice versa.

## Methods

The posteroanterior and lateral spinal radiographs of 83 referred girls from the "Thriasio" school screening program were evaluated in order to determine the influence of age in the correlation between the rib cage and the spinal deformity. The mean age of the examined girls was 13.4 years (range 7–18). All had trunk asymmetry ≥ 7° in any of the three examined regions of the spine (thoracic, thoracolumbar, or lumbar), as it was measured by the scoliometer and expressed as Angle of Trunk Inclination (ATI).

**Figure 1:**
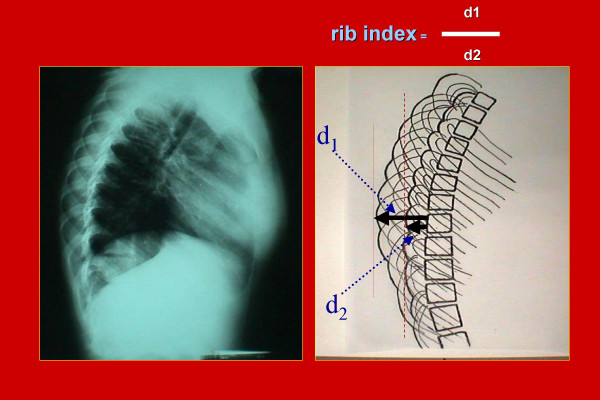
A drawing of a lateral spinal radiograph describing the rib-index. The rib-index is the ratio d1/d2. d1 is the distance between the posterior margin of the vertebral body and the most extended point of the most projecting rib contour. d2 is the distance between the posterior margin of the same vertebral body and the most protruding point of the least projecting rib contour.

The spinal deformity was assessed by measuring the Cobb angle from the postero-anterior spinal radiographs. The rib cage deformity was quantified on the lateral spinal radiographs by measuring the rib-index at the apex of the thoracic curve. The rib-index is defined as the ratio of two distances (d1/d2). The first (d1) is the distance between the posterior margin of the vertebral body and the most extended point of the most projecting rib contour, while the second (d2) is the distance between the posterior margin of the same vertebral body and the most protruding point of the least projecting rib contour [20], (Figure 1).

The normality of the data was verified with the Shapiro-Wilk test for normal data. No variable deviated from normal distribution. The magnitude of the correlation between the dependent variable "Cobb angle" and the independent variables "rib index" and "age" was estimated by calculation of Pearson's correlation coefficients. After calculating the median age, we divided our sample in two subgroups, consisting of younger and older participants. Separate univariate linear regression analysis was then performed for each age subgroup in order to assess the effect of age on Cobb angle and rib index correlation. The overall significance of the models was based on the calculation of F statistic. All statistics were two-sided and considered significant if p-value was less than 0.05. Analysis was performed using STATA™ (Version 9.0, Stata Corporation, College station, TX 77845, 800-782-8272).

## Results

Fourteen out of the 83 girls had straight spines. Seven were found with a curve less than 10°, while 31 had thoracic curves, 10 had thoracolumbar curves and 21 had lumbar curves. The descriptives of the examined girls are shown in Table 1.

**Table 1:** Descriptives of the age, the rib-index, the thoracic, thoracolumbar and lumbar Cobb angle of the examined girls (n = 83) with different curve types

	***Minimum***	***Maximum***	***Mean value***	***Standard Deviation***	***Curve type***
Age (years)	7	18	11.6	3.7	**Straight spines (n = 14)**
Rib-Index	1.2	2.3	1.4	0.3	
Thoracic Cobb	0°	0°	0°	0°	
Thoracolumbar Cobb	0°	0°	0°	0°	
Lumbar Cobb	0°	0°	0°	0°	

Age (years)	7	16	12.4	3.1	**Curves < 10**° **(n = 7)**
Rib-Index	1.3	1.9	1.6	0.2	
Thoracic Cobb	0°	9°	3.6°	4.5°	
Thoracolumbar Cobb	0°	8°	1.2°	3°	
Lumbar Cobb	0°	8°	3.4°	4.3°	

Age (years)	8	17	14.1	2.6	**Thoracic Curves (n = 31)**
Rib-Index	1.1	2.2	1.6	0.3	
Thoracic Cobb	10°	22°	14°	2.9°	
Thoracolumbar Cobb	0°	11°	0.4°	1.98°	
Lumbar Cobb	0°	18°	4.5°	6°	

Age (years)	11	18	14.5	1.8	**Thoracolumbar curves (n = 10)**
Rib-Index	1.2	2.3	1.6	0.3	
Thoracic Cobb	0°	11°	1.1°	0.4°	
Thoracolumbar Cobb	10°	17°	12.9°	2.6°	
Lumbar Cobb	0°	8°	1°	2.5°	

Age (years)	8	17	13.2	2.2	**Lumbar curves (n = 21)**
Rib-Index	1	2.4	1.5	0.3	
Thoracic Cobb	1°	12°	3.4°	4.7°	
Thoracolumbar Cobb	0°	0°	0°	0°	
Lumbar Cobb	10°	24°	15°	4°	

The correlation between the dependent variable "Thoracic Cobb angle" and the independent variable "rib-index" without adjusting for age was not statistically significant (Pearson's correlation coefficient r = 0.197, p = 0.077). This was also the case for the correlations between Thoracolumbar Cobb angle and rib-index and between lumbar Cobb angle and rib-index (r = 0.105, p = 0.350, r = 0.052, p = 0.642, respectively), Table 2.

**Table 2:** Pearson's correlation coefficients (r) between the dependent variable "Cobb angle" and the independent variable "rib-index" with and without the effect of the (predictor) variable "age"

	**"Thoracic Cobb angle"**	**"Thoracolumbar Cobb angle"**	**"Lumbar Cobb angle"**
"Rib-index" without the effect of "age"	r = 0.197 p = 0.077	r = 0.105 p = 0.35	r = 0.052 p = 0.642

**Figure 2:**
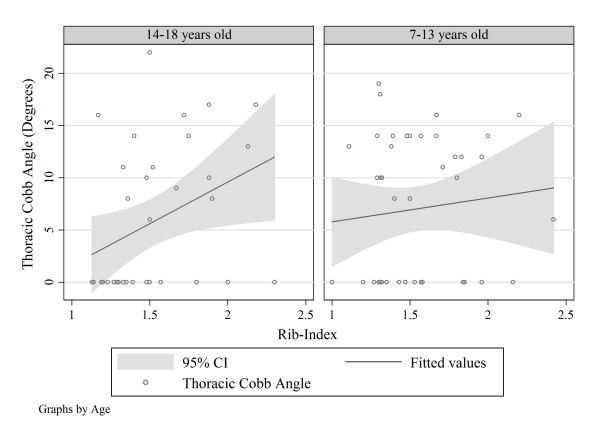
The only linear association was the one between Thoracic Cobb Angle and rib-index in the age group of 14–18 years. (*Predicted Thoracic Cobb Angle = - 6.357 + 7.974 × (Rib-Index)*.

After calculating the median age (14 years), two subgroups were created; group A (7–13 years old, 37 subjects, 44.58%) and group B (14–18 years old, 46 subjects, 55.42%). Following the split, the results of the univariate linear regression models of various Cobb angles and rib index for each age group, are presented in Table 3. The only linear association was the one between Thoracic Cobb Angle and rib-index in the age group of 14–18 years (Predicted Thoracic Cobb Angle = -6.357 + 7.974*(Rib-Index). The linear relationship between Thoracic Cobb angle and rib-index is shown graphically in (Figure 2).

**Table 3:** Univariate linear regression models by age group. Thoracic Cobb angle, Thoracolumbar Cobb angle and Lumbar Cobb angle are the dependent variables. Rib-index is the independent variable

**Dependent variable**	**Age group**	**Explanatory variable**	**Unstandardized beta coefficient**	**Standard Error**	**t**	**p-value**	**[95% Conf. Interval]**	
Thoracic Cobb angle	7–13	Rib-index	2.283	3.369	0.680	0.502	-4.520	9.086
		Constant	3.496	5.306	0.660	0.514	-7.221	14.212
Thoracic Cobb angle	14–18	Rib-index	7.974	3.524	2.260	0.030	0.805	15.144
		Constant	-6.357	5.474	-1.160	0.254	-17.494	4.781
								
Thoracolumbar Cobb angle	7–13	Rib-index	-0.946	0.902	-1.050	0.302	-2.782	0.890
		Constant	1.724	1.402	1.230	0.227	-1.128	4.576
Thoracolumbar Cobb angle	14–18	Rib-index	3.116	2.827	1.100	0.277	-2.594	8.826
		Constant	-1.605	4.454	-0.360	0.720	-10.600	7.390
								
Lumbar Cobb angle	7–13	Rib-index	0.804	4.229	0.190	0.850	-7.799	9.408
		Constant	6.320	6.569	0.960	0.343	-7.045	19.684
Lumbar Cobb angle	14–18	Rib-index	1.901	3.252	0.580	0.562	-4.666	8.469
		Constant	1.876	5.123	0.370	0.716	-8.469	12.222

## Discussion

The detection of spinal deformities through the various screening programs is a challenging issue. Initially the forward bending test and later the use of back shape analysis methods, such as scoliometer and Moire topography were followed by an increased number of false positive results and an increased number of referrals and unnecessary radiographs [17]. The more advanced 3-D computer assisted systems and the various body scanners are quantifying more accurately the surface morphology of the trunk and efforts have been made to correlate these findings with the spinal deformity.

The present study shows that in younger children the concordance of the surface and spinal deformity is weak and it becomes stronger as the children are growing up. Therefore, in younger children with surface trunk asymmetry, the prediction of the spinal deformity alone from the surface topography is inaccurate, simply because surface topography reveals the thoracic cage and the spinal deformity together. Furthermore the Cobb angle alone cannot explain the whole of the surface deformity [10]. Fourteen out of 83 girls (16.9%) in our study had straight spines, although the scoliometer readings were ≥ 7°. When adding the 7 girls with spinal curves <10°, it is interesting that 21 girls (25%) with an ATI ≥7° had a spinal curve under 10° or had a straight spine.

The rib-index clearly demonstrates the thoracic cage deformity and when its value is above 1, it displays the existence of surface asymmetry, which is the main indicator for referral during school screening for scoliosis [20]. The rib-index is a radiological sign and thus it is not obtainable by the screening programs, but is more meaningful when studying the correlation between the surface and the spinal deformity.

The role of the rib cage in the pathogenesis of idiopathic scoliosis has been implicated in the past [21-27].

The growth of the thoracic spine and the growth of the rib cage are directly related and that a growth disturbance of one induces deformity in the other [28]. Either unilateral rib or spine tethering produces both a scoliosis and rib cage deformity [29]. The deformity induced by unilaterally tethering the ribs is much greater than the deformity induced by unilaterally tethering the transverse processes of the spine. This may be a consequence of the longer moment arm provided by the ribs, thereby producing a larger bending moment to deform the thoracic spine [28].

The spine and ribs work together efficiently at respiration as a dynamic biomechanical structure only under specific conditions [30]. When the thorax is affected by significant deformity, the dynamics of this system change, interfering with normal respiration and lung development [28]. Sevastik et al induced scoliosis experimentally in young New Zealand rabbits either by performing rib osteotomies and interposing a metallic ring into the osteotomy gap to asymmetrically elongate the ribs or by unilaterally segmenting three intercostal nerves [31,32]. In addition, abnormalities in the evolution of anterior chest wall blood supply were implicated in the pathogenesis of progressive right-convex female thoracic scoliosis [27]. On the contrary, young children suffering thoracic insufficiency syndrome and undergoing spine fusion for scoliosis may continue to develop significant thoracic hypoplasia, restrictive lung disease and respiratory insufficiency by early adulthood with early death [30]. A clearer understanding of this reciprocal association between the growth of the rib cage and the thoracic spine has never been quantified. The findings of the present study, which includes mild scoliotic curves, correlate the growth of the rib cage and the thoracic spinal deformity, supporting the hypothesis that the rib cage deformity precedes the spinal deformity in the pathogenesis of idiopathic scoliosis, but can not exclude that pathogenesis might be in the vertebral column.

Age is a very important factor and has a definite effect, since it influences the correlation between the surface and the spinal deformity. In younger children this correlation is very weak, while it is stronger in older children. This important finding of the existence of remarkable rib cage deformity without simultaneous spinal deformity in younger school screening referrals requires further research. A longitudinal study ought to be conducted to discriminate the percentage of children that will in time develop scoliosis and the possible responsible factors.

As a result of the effect of growth on the correlation between the thoracic surface deformity and the spinal deformity, the predictive value of the existing formulas which calculate the Cobb angle from surface measurements is poor. Therefore our recommendation is to take into consideration the effect of growth when developing such predictive models, otherwise they can be inaccurate.

One more interesting outcome from this study is that screening younger children for scoliosis is beneficial, at least for the purpose of scoliosis aetiology research. This study could not be completed and the above findings couldn't be resulted unless younger children were screened in our scoliosis school screening program.

These findings may also have implications for the conservative treatment in younger scoliotics with braces when indicated. The correction of the more pronounced rib cage deformity which is addressed by the brace could easily prevent the deterioration of the less deformed "central axis" that is the spinal column at an earlier stage.

In conclusion growth seems to have a significant effect in the correlation between the rib cage and the spinal deformity in girls with IS. The findings of the present study support the hypothesis that the correlation between thoracic surface and spinal deformity is weak in younger children, implicating that the thoracic cage deformity precedes that of the spine in the pathogenesis of idiopathic scoliosis.

## Authors' contributions

TBG conceived the idea of the study, performed part of the literature review, performed the statistical analysis, interpreted the data and contributed in drafting of the manuscript. EV contributed in manuscript drafting and in the interpretation of data, performed part of the literature review and part of the statistical analysis. CM contributed in the statistical analysis. The authors have read and approved the final manuscript.
